# Whole-Genome Shotgun Sequence of *Bacillus amyloliquefaciens* Strain UASWS BA1, a Bacterium Antagonistic to Plant Pathogenic Fungi

**DOI:** 10.1128/genomeA.00016-14

**Published:** 2014-02-06

**Authors:** F. Lefort, G. Calmin, P. Pelleteret, L. Farinelli, M. Osteras, J. Crovadore

**Affiliations:** a Plants and Pathogens Group, Research Institute, Earth Nature and Environment, Hepia, University of Applied Sciences of Western Switzerland, Geneva, Switzerland; b Faculty of Engineering and Architecture, University of Applied Sciences of Western Switzerland, Delémont, Switzerland; c Fasteris SA, Plan-les-Ouates, Geneva, Switzerland

## Abstract

We report here the whole-genome shotgun sequence of *Bacillus amyloliquefaciens* strain UASWS BA1, isolated from inner wood tissues of a decaying *Platanus* × *acerifolia* tree. This strain proved to be antagonistic to several plant pathogenic fungi and oomycetes and can be developed as a biological control agent in agriculture.

## GENOME ANNOUNCEMENT

*Bacillus amyloliquefaciens* Fukomoto 1943, redescribed by Priest et al. (1), has been extensively used for producing α-amylase and proteases for industrial uses (2). Some strains were identified as antibiotic producers, plant growth promoters, and plant health enhancers, and then were used as biocontrol agents. Plant growth-promoting rhizobacterial (PGPR) strains of *B. amyloliquefaciens* were distinguished as *B. amyloliquefaciens* subsp. nov. *plantarum* (3) by genome comparison and classical bacterial taxonomy, while other strains were considered to be *B. amyloliquefaciens* subsp. nov. *amyloliquefaciens*. The available complete genome sequences showed variation in their genome sizes, from 3.86 Mb up to 4.24 Mb (4–9), with a G+C content range of 45.7 to 46.6%. Plant-associated *B. amyloliquefaciens* strains usually display antifungal and antibacterial activities mediated by secondary metabolites, such as lipopeptides and polyketides (7–9). The strain *B. amyloliquefaciens* UASWS BA1 was isolated in Geneva from inner symptomatic wood samples of a decaying *Platanus* × *acerifolia* tree. DNA was purified from axenic isolates according to a modified DNA extraction micromethod (10). Whole-genome shotgun sequencing was then performed in an IIlumina HiSeq 2000, producing 26,331,262 paired-end reads 100 bp long. Assembly was carried out with ABySS 1.3.5 (11) and yielded 24 scaffolds for a genome length of 3,944,088 bp, with a G+C content of 46.6% and a scaffold N_50_ value of 346,879 bp. It included a plasmid of 8,106 bp in length. Annotation was realized upon submission through the NCBI Prokaryotic Genomes Automatic Annotation Pipeline (PGAAP) and allowed for the identification of 113 RNA genes (29 rRNA and 84 tRNA genes). Genome comparisons in RAST 4.0 (12) identified the closest neighbors as *B. amyloliquefaciens* strains LL3 (13), XH7 (14), IT45, and FZB42 (5). RAST 4.0 analysis also identified 3,980 coding sequences (CDSs), 47% of which were allocated a function. Missing genes were estimated to be about 54. It also confirmed the presence of nonribosomal peptide synthetase (NRPS) gene clusters encoding surfactin production (*comS*, *srfA*, *rapA*), genes for bacilysin (*bacA*, *bacB*, *bacE*) and a bacillibactin siderophore, and several polyketide synthase (PKS) genes. The bacterium was equipped for resistance to drugs and heavy metals, aromatic compound degradation, motility, and chemotaxis and for the synthesis of auxin precursors probably useful in the symbiotic relationship with plants. It also harbors genes for capsule exopolysaccharides and endospore proteins. Extended studies and comparisons of the genome of *B. amyloliquefaciens* UASWS BA1 would enable the elucidation of the mechanisms supporting its antifungal properties, which would make this strain a potential biocontrol agent.

### Nucleotide sequence accession numbers.

This whole-genome shotgun (WGS) project was deposited at GenBank under the accession no. AWQY00000000. The version described in this paper is the first version, AWQY00000000.1 (Genbank assembly ID GCA_000469015.1; RefSeq assembly ID GCF_000469015.1).
